# On-Channel Integrated Optofluidic Pressure Sensor with Optically Boosted Sensitivity

**DOI:** 10.3390/s19040944

**Published:** 2019-02-23

**Authors:** Noha Gaber, Ahmad Altayyeb, Sherif A. Soliman, Yasser M. Sabry, Frédéric Marty, Tarik Bourouina

**Affiliations:** 1 Center for Nanotechnology, Zewail City of Science and Technology, October Gardens, 6th of October, Giza 12578, Egypt; aaltayeb@zewailcity.edu.eg (A.A.); s-sherif.soliman@zewailcity.edu.eg (S.A.S.); 2 Université Paris-Est, ESIEE Paris, ESYCOM EA 2552, 93162 Noisy-le-Grand, France; frederic.marty@esiee.fr (F.M.); tarik.bourouina@esiee.fr (T.B.); 3 Faculty of Engineering, Ain-Shams University, Cairo 11566, Egypt; yasser.sabry@eng.asu.edu.eg

**Keywords:** Fabry–Pérot cavity, optical pressure sensor, integrated microresonator, Bragg mirrors

## Abstract

A novel optofluidic sensor that measures the local pressure of the fluid inside a microfluidic channel is presented. It can be integrated directly on-channel and requires no additional layers in fabrication. The detection can be accomplished at a single wavelength; and thereby, only a single laser diode and a single photodetector are required. This renders the sensor to be compact, cheap and easy to fabricate. Basically, the sensor consisted of a Fabry–Pérot microresonator enclosing the fluidic channel. A novel structure of the Fabry–Pérot was employed to achieve high-quality factor, that was essential to facilitate the single wavelength detection. The enhanced performance was attributed to the curved mirrors and cylindrical lenses used to avoid light diffraction loss. The presented sensor was fabricated and tested with deionized water liquid and shown to exhibit a sensitivity up to 12.46 dBm/bar, and a detection limit of 8.2 mbar. Numerical simulations are also presented to evaluate the mechanical–fluidic performance of the device.

## 1. Introduction

Pressure microsensors are widely employed in several applications, such as the automotive industry, for engine control systems or tire pressure monitoring [1]; biomedical applications for measuring blood pressure, or intraocular eye pressure [2]; and aerospace applications in modem aircrafts for engine and hydraulics control, or environmental control [3]. Pressure measurement along the fluidic microchannel is essential in lab-on-chip systems, as it controls the flow rate and movement of the solvents and samples, or even handling and separating the biological cells [4]. It also can be used to infer the mechanical properties of the sample [5]. Several on-channel pressure sensors for local pressure measurements have been developed, based on optical [6,7,8], or electrical methods [9]. Optical methods are appealing for their high sensitivity, immunity to electromagnetic interference, and easy integrability. However, the requirement for large-scale instruments, such as frequency-selective equipment or imaging equipment, besides the complicated analysis steps, hinder their appeal in some applications. Widespread optical sensors rely on the change in the resonant wavelength, such as those based on Bragg fiber gratings [10], Fabry–Pérot (FP) resonators [6,8], or ring resonators [11]. These resonators are widely used as essential micro-optical components and can be exploited in many other applications, besides pressure sensing, such as optical trapping [12,13], refractometry [14,15], biological cells detection [16], and temperature sensing [10]. To record the spectrum and locate the shift in resonance, either a spectrometer or a tunable laser is required. There have been some trials for integrating spectrometers, based on tunable filters [17,18], but they are sophisticated and expensive to fabricate as they require multistep and various fabrication processes. Also, they require tuning or scanning, in addition to post-measurement calculations.

Another category of sensors is based on spatial interferometry [7,19], which requires an imaging camera to record the interference pattern. Although such a scenario is less bulky and expensive than the first one, it is still not easy to produce a good image in a tight space, and special imaging optics are usually required. Also, certain separation distances—sometimes in the millimeter and centimeter range—are required for the beam to spread before reaching the camera, so that the periodicity of the interference fringes get larger than the pixels’ pitch in order to be resolved. Therefore, a pressure sensor based on measuring only the intensity is preferred to not hinder the miniaturization of the sensor. It is worth mentioning that the Mach–Zehnder interferometer can also achieve a single readout [20]. However, it is polarization dependent and its size is in the centimeter range, which occupies a very large area on the chip. The novel technique proposed in this work is implemented on the microfluidic channel directly and requires measuring only a single optical power value, which requires a single photodiode. This avoids the above-mentioned complications, besides achieving the measurement at very high-speed in almost real-time, with no scanning or data analysis required.

Another important issue is the fabrication technique and enabled wafer-level integration with the microfluidic chip. The vast majority of miniaturized pressure sensors rely on altering the optical fiber itself to be the sensor [8,21,22,23], this is very suitable for the in vitro pressure measurement to reach the fluid inside the body [23]. However, for on-chip measurement, such a feature is not the main interest; compatibility with fabrication processes is far more desired. Therefore, a pressure sensor fabricated in patch-processing along with the chip itself is preferred over the non-standard fabrication process of altering the optical fiber. Also, the configuration of the sensor with respect to the microfluidic channel is important. Some pressure sensors are placed perpendicular to the chip surface [24,25]. This off-plan configuration might be inconvenient to some users—to not hinder the channel viewing for instance—besides, the fiber fixation may not be very stable. For those later users, the proposed in-plan fiber connection may be more appropriate. Some pressure sensors for microfluidic devices require the construction of a side channel for measurement [24], which consumes area and complicates the design. This is not the case in our novel design, as it is directly on-channel, with no much additional area occupied by the sensor.

In this article, the implementation of a novel optical pressure sensor is presented that overcomes the above-stated challenges. The introduced method depends on a single readout of optical power at a single wavelength, and hence can provide real-time measurement with no scanning or post-processing. Depending on a single wavelength requires only a single laser diode and a single detector, which makes it cheap and easy to integrate. Also the structure was simple and easily fabricated with a single mask. In what follows, a detailed description of the device is illustrated; then, numerical simulation of the mechanical–fluidic interaction is performed; and finally, the experimental fabrication and characterization are explained. The effect of various design parameters is discussed along with the achieved measurements by different structure dimensions.

## 2. Materials and Methods

### 2.1. Proposed Structure

**Figure 1:**
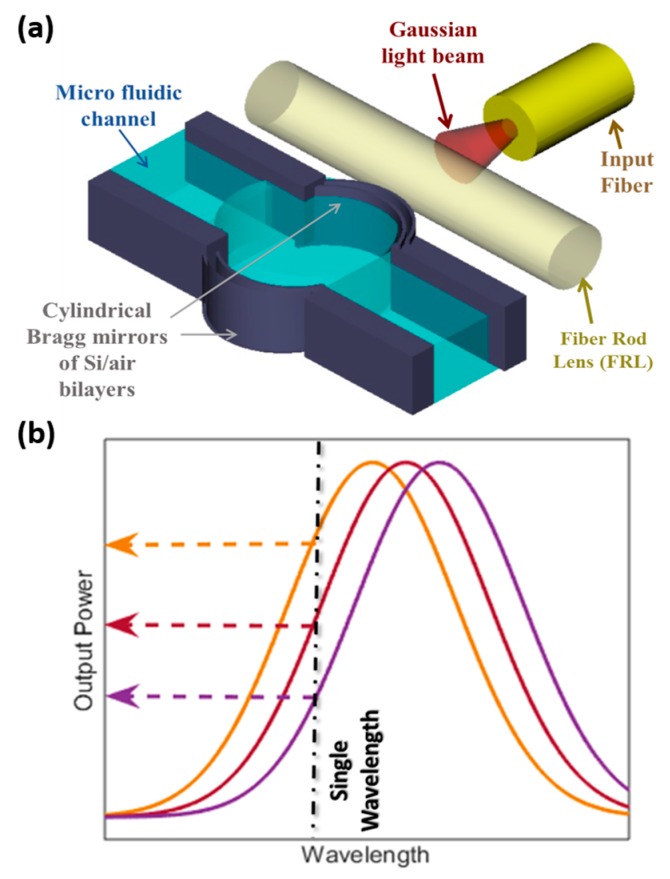
(**a**) Schematic of the proposed on-channel optical pressure sensor; (**b**) Schematic indicates the new measurement method of the power level measurement at a single wavelength.

The proposed structure is presented in Figure 1a. It was based on an FP optical resonator consisting of two cylindrical Bragg mirrors, enclosing the microfluidic channel, holding the flowing liquid. Each Bragg mirror consisted of multi silicon/air bilayers. The thicknesses of the silicon layers were 3.67, and the air gap thickness was 3.49 µm. These thicknesses corresponded to multiples of a quarter of the central wavelength, of 1550 nm, inside each medium. The cylindrical mirrors had a radius of curvature of 140 µm. This curvature provided confinement of light in the in-plane direction (parallel to the substrate), which helped to increase the quality factor of the micro-optical FP resonator, as previously demonstrated theoretically and experimentally by our group [14]. For further out-of-plan light confinement (in the direction perpendicular to the substrate), an external cylindrical rod lens was placed horizontally at the cavity entrance. This lens is a fiber rod lens (FRL) had a diameter of 125 µm, like the fiber used for delivering the input light beam, which ensured that the beam passed through its diagonal. Such FP with a curved surfaces design can achieve quality factors over 1800, as will be demonstrated by the experimental work. This was much higher than that achieved by straight mirror FP microresonators, which are usually in the orders of several hundred [16,26], and close to what is achieved by nanoring resonators [27]. Of course, microring resonators achieve higher quality factors, that range from a few thousand to hundreds of thousands, but this comes on the expense of the fabrication complexity and cost, as they require different materials for core and cladding. So, either deposition is adopted, which is difficult to control due to stresses [28]. Alternatively, expensive Silicon-On-Insulator (SOI) wafers can be employed [29]. In addition, it is challenging to couple light into these resonators and special evanescent field methods are commonly used.

The proposed FP microresonator with curved surfaces can be used for pressure sensing, as when the fluid passes through the fluidic channel, it exerts pressure on its walls. Therefore, the cylindrical cavity walls get deformed according to the value of the exerted pressure. The mirror deformation causes the cavity length to change by the deformed amount on both sides, and as the optical resonance happens only when the cavity length equals multiples of half the wavelength, then the optical resonance spectrum shifts to fit this condition. Figure 1a schematically depicts the spectral shift in the resonance peaks for different pressures. Usually in the literature, the signal is extracted from measuring the spectral shift [6,7], and hence, the whole spectrum should be recorded. This requires instruments, such as optical spectrum analyzers or tunable lasers, which complicate the measurement setup. Overcoming that, the method we propose employs measuring the output power at a single wavelength only, as indicated in Figure 1b. This can be achieved thanks to the high-quality factor of the adapted FP resonator design, such change in power can express the pressure directly with no tedious post-processing for the data. The total sensitivity of the device (S) is then the change in output optical power (ΔW) with the change of fluidic pressure (ΔP). It is equal to the product of mechanical sensitivity and optical sensitivity. Mechanical sensitivity is the change in cavity length (ΔL) per change of pressure, while optical sensitivity is the change of optical power per change of cavity length. This is expressed by Equation (1):

(1) $$S=\frac{\Delta W}{\Delta P}=\frac{\Delta W}{\Delta L}\cdot \frac{\Delta L}{\Delta P}$$

### 2.2. Numerical Simulation

**Figure 2:**
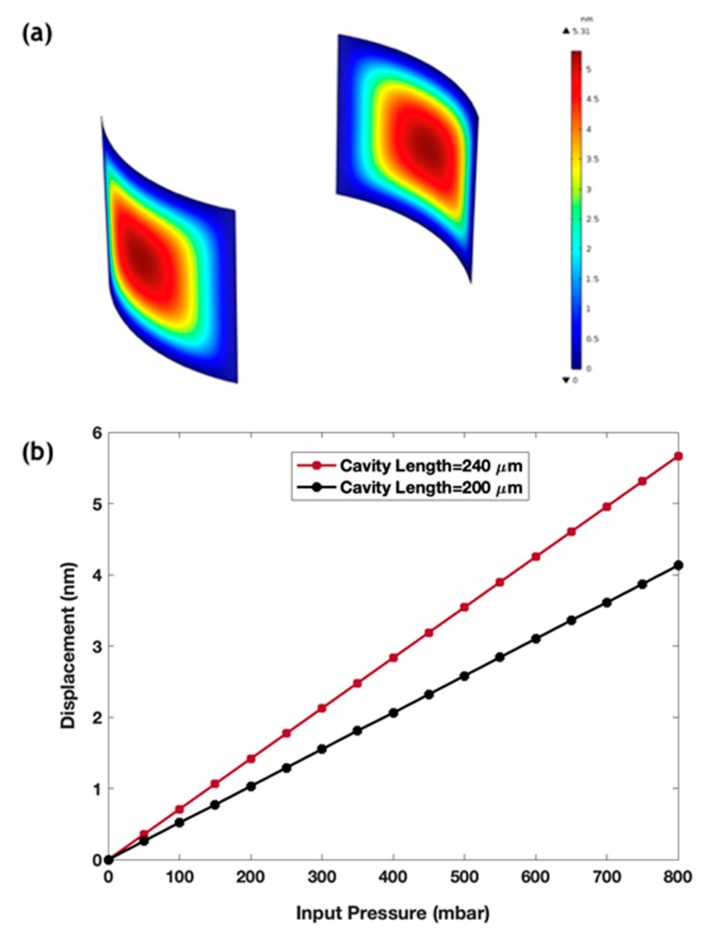
(**a**) The finite element method (FEM) simulation of the deformation in the cavity walls caused by a fluid pressure of 800 mbar inside a cavity of 240 µm length; (**b**) Maximum deflection at different pressure values for two cavities of different lengths of 200 µm and 240 µm.

The finite element method (FEM) simulation by the software package COMSOL multiphysics was used to model the interaction of the fluid inside the sensor with the curved mirror walls. The fluid–structure interaction interface, which models two-way coupling between solids and fluids, has been used to solve the Stokes equations for the conservation of momentum and the continuity equations for conservation of mass. An Arbitrary Lagrangian–Eulerian (ALE) formulation was used for incorporating the geometrical changes of the fluid domain. The fixed constraint boundary condition was applied to the mirror walls that were not in contact with the fluid. To solve the above system of equations, we used the fully coupled GMRES linear solver to solve for the velocity, pressure and displacement fields. The parametric sweep feature in the COMSOL study was used to solve for different externally applied pressures. A relative tolerance of 10^−3^ was used as a convergence criterion. Figure 2a presents the distribution of the displacement of the curved silicon walls when a liquid flowed into the channel inside a cavity of 240 µm length at an input pressure of 800 mbar at the input port. Figure 2b plots the maximum deflection at different pressures, from which, a mechanical sensitivity can be estimated from the slope of the linear graph. Mechanical sensitivity of 7.08 nm/bar was achieved for a cavity of length 240 µm, while it was 5.16 nm/bar for a cavity of length 200 µm. It should be noted that the total sensitivity of the device depends also on the optical sensitivity, which was boosted by our optical detection method, as will be demonstrated in the results section. 

Some pressure sensors in the literature aim to enhance mechanical sensitivity by employing flexible polymer membranes to get high deflection [6,7,8]. Our membranes are the first layer of the silicon Bragg mirrors. Although silicon is less flexible than polymer, a thin layer of only 3.7 µm achieved acceptable deflection values that could be resolved by our detection technique; while using silicon rendered the sensor fabrication compatible with the standard silicon technology. Also, in our sensor, the mechanical sensitivity was doubled as the deformation happened in the two walls of the FP cavity, while almost all sensors in the literature allow only one wall to deform. 

## 3. Experimental

**Figure 3:**
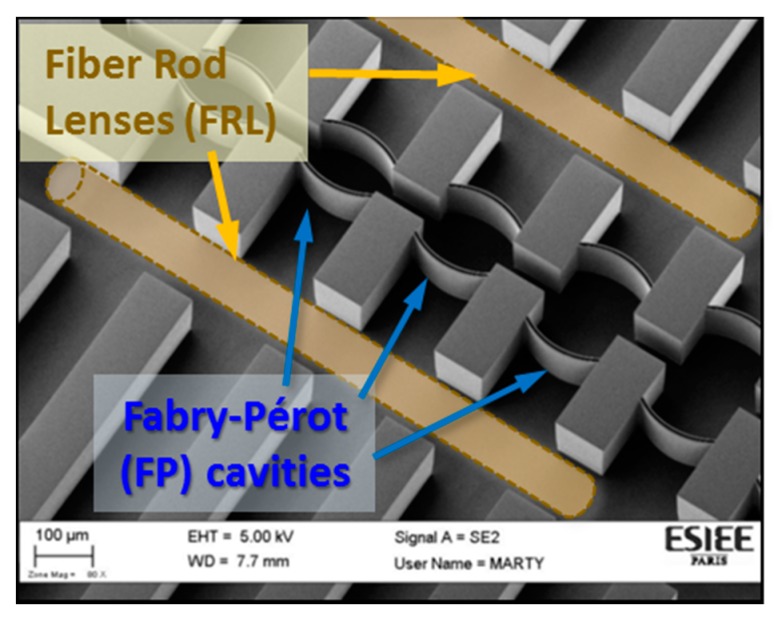
SEM image of the fabricated chip, and the inserted fiber rod lenses (FRLs).

The structure was fabricated by standard silicon microfabrication techniques inside a class 100 cleanroom. Starting with a single-crystalline p-type silicon with (100)-orientation, a hard mask of 400 nm thick thermal oxide was created. The pattern was then etched into bulk silicon by employing the Bosch process of the deep reactive ion etching (DRIE) technique. This etching step was critical, as very smooth and vertical walls were required for the Bragg mirror to minimize the optical losses. It has been optimized for producing such demanding requirements, along with high etching depths into silicon that exceeds 130 µm. As the grooves in the chip host the FRLs and the measuring fibers, whose diameters are about 125 ± 3 µm, it is understandable why such a high depth was essential for accommodating these fibers with enough clearance for easy implementation. A scanning electron microscope (SEM) image of the structure is presented in Figure 3. Multi cavities with different lengths and different numbers of silicon/air bilayers were implemented on the very same chip. This provided different quality factors and free spectral ranges of the resonators, that affected the sensitivities and measuring ranges for the sensor, as will be demonstrated in the results section. After fabrication and dicing of the silicon die, they were capped by pieces of cured polydimethylsiloxane (PDMS). Their bonding was done by activating the two surfaces in oxygen plasma, then pressing them together. Finally, the FRLs made by pare fibers were placed into their grooves. For connecting the fluidic input/output ports, holes in the PDMS were pinched to insert capillary tubes of 360 µm diameter, that fit the micro-size dimensions ports, on the chip. Then a standard tubing was used to connect the liquid reservoir and the pumping system. In this work, deionized (DI) water was used as a test liquid, and it was pumped with a controlled pressure using the pressure controller MFCS™-EZ from Fluigent. The optical measurement setup consisted of a tunable laser source covering the L and C bands of the near infrared and a power meter, along with a photodetector head. Both were controlled by a computer for achieving synchronized scanning of the laser wavelength with the corresponding measured power. Single mode cleaved fibers were used for injecting and collecting the light into and from the chip.

## 4. Results and Discussion

Various FP cavities with different dimensions and design parameters were fabricated together on the silicon chip, as demonstrated earlier. The longer cavity length (L) provides a higher quality factor (Q) as stated by Equation (2):

(2) $$Q=\frac{2\pi nL\sqrt{R}}{1-R}$$

where n is the refractive index of the fluid inside the cavity, and R is the mirror reflectivity. The higher quality factor rendered the resonance peaks more sharp with steeper sides, which achieved higher sensitivity. The reflectivity of the Bragg mirror increased by increasing the number of silicon/air bilayers, which increased both the quality factor and the contrast between the maxima and minima power value. This was favorable for both sensitivity and range, but lowered the transmitted power, which may have rendered the measurement of the transmission spectra more difficult and vulnerable to noise. In what follows, two cavities with different dimension were tested as pressure sensors to validate the effects.

### 4.1. First Cavity

**Figure 4:**
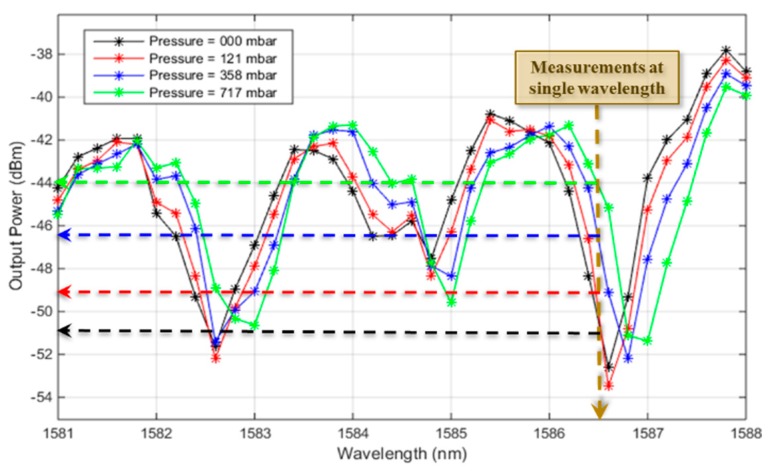
Measured transmission spectra from the Fabry–Pérot (FP) cavity at different pressure values.

This cavity had a physical length of 200 µm and its Bragg mirrors consisted of two bilayers. The transmission spectra were recorded in the beginning as a calibration step, only to determine the optimal wavelength of operation. Figure 4 plots the measured spectra for different pressure values exerted by the pressure controller. From these spectra, the single wavelength of operation was selected at 1586.5 nm, as indicated by the brown line in Figure 4. Such a wavelength was chosen to be located in the linear region of the side of the resonance peak, and when the pressure changed, the chosen wavelength still fell in the linear region but at different power values. The selected resonance peak had a quality factor of about 1495. It is worth mentioning that the best resonance performance deviated slightly away from 1550 nm due to a slight change in dimensions after the fabrication of such a challenging high-aspect-ratio structure. 

**Figure 5:**
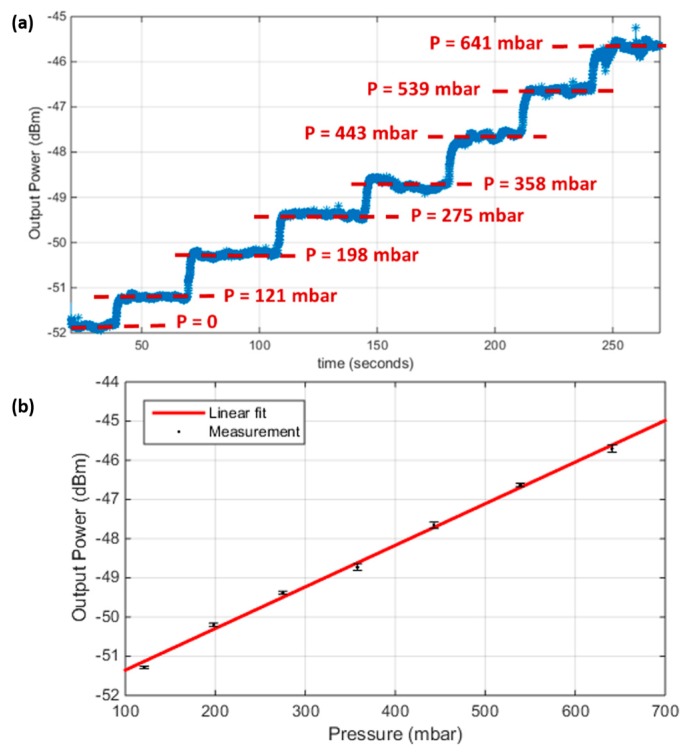
(**a**) The output power signal change with changing the pressure versus time; (**b**) The output power signal versus the applied pressure, for the second cavity of length = 200 µm and Bragg mirrors of 2 bilayers.

After that calibration, the light source was fixed at the selected wavelength. The pressure was changed from 0 to 641 mbar in steps along an interval of time of about 30 s, and the corresponding optical power was recorded. Figure 5a shows the recorded power values upon changing the pressure along the time, while injecting a single wavelength into the cavity. The optical signal at each pressure step was analyzed to obtain the average and root mean square (rms) error values. These data are plotted in Figure 5b. The points are the average values and the error bars represents the rms error. The total sensitivity corresponds to the slope of the linear plot in Figure 5a, and was obtained to be about 10.614 dBm/bar. The range was only limited by our test equipment and could exceed 700 mbar. The resolution of a sensor is estimated by three times the root mean square error value due to the noise variations, which is the standard deviation (*σ_e_*). From the error bars in Figure 5b, the maximum standard deviation was about 0.093 dBm, then the resolution was 3 *σ_e_* = 0.279 dBm. The detection limit (DL) is the smallest change in pressure that can be accurately detected, and is equivalent to the resolution, but in the pressure units transformed by the sensitivity. From the above-stated values, the DL was estimated to be about 26.3 mbar. It is worth noting that the method of tracing the optical power could resolve different pressure values with a step smaller than the ordinary method of tracing the resonance wavelength peak. One can notice from the spectra in Figure 4, that it was difficult to accurately identify the peak wavelength value, even for a large pressure difference, due to the poor step of the scanned wavelength. Of course, a more accurate identification could be provided by more sophisticated equipment with a smaller wavelength step, but they will of course, be more expensive and difficult to integrate on-chip. 

### 4.2. Second Cavity

**Figure 6:**
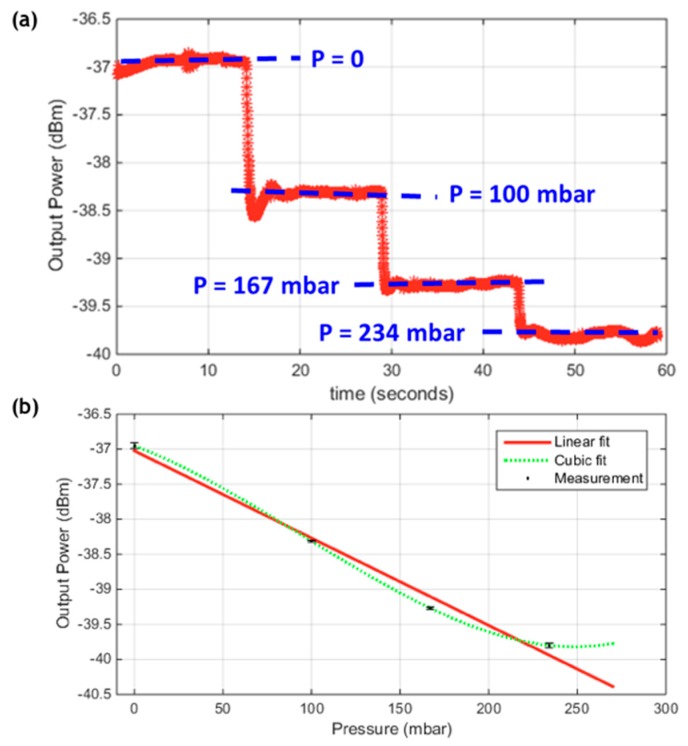
(**a**) The output power signal change with changing the pressure versus time; (**b**) The output power signal versus the applied pressure, for the second cavity of length = 240 µm and Bragg mirrors of 5 bilayers.

Another cavity of a physical length of 240 µm and Bragg mirrors of 5 bilayers was tested. This cavity gave a higher quality factor of about 1812, and hence a higher sensitivity for smaller changes. However, the range of pressures that could be sensed within the linear region became more limited. Figure 6a displays the output optical power with changing the pressure along the time, and Figure 6b displays that optical power signal versus pressure values after analyzing the recorded data in Figure 6a and obtaining the average and the rms error. The overshoots at the transient from one pressure value to another were verified to be from brief instability in the pressure controller, until it reached the steady-state at the prescribed value, not from our optical sensor. The sensitivity, in this case, was 12.46 dBm/bar, which was higher than the previous one. But on the other hand, the range was much reduced. As observed in Figure 6b, the measured points could hardly be fit by a linear trend, and a cubic fit was much better. This revealed that linear region of the side resonance peak was exceeded by the few last points and probably were located near to a summit of the resonance peak. The linear range could be estimated by about 180 mbar in this case. The standard deviation for this case was about 0.034 dBm, and thereby, the resolution and DL were 0.102 dBm and 8.2 mbar, respectively.

## 5. Conclusions

We demonstrated a novel method for on-channel local pressure measurement by detecting only a single optical power value. The device was thus more compact and cheap, and suitable for fast real-time measurements. The optical sensitivity achieved by this method depended on the slope of the spectral peaks of the FP resonator, and hence its quality factor. Therefore, a high-quality factor was required, which was achieved by curved mirrors and lenses. The long cavity lengths also increased the quality factor and hence achieved higher sensitivity, but limited the measurement ranges. Therefore, a compromise in performance, according to the application, should be made. And accordingly, the structure dimensions should be designed.
